# Additional Telemedicine Rounds as a Successful Performance-Improvement Strategy for Sepsis Management: Observational Multicenter Study

**DOI:** 10.2196/11161

**Published:** 2019-01-15

**Authors:** Robert Deisz, Susanne Rademacher, Katrin Gilger, Rudolf Jegen, Barbara Sauerzapfe, Christina Fitzner, Christian Stoppe, Carina Benstoem, Gernot Marx

**Affiliations:** 1 Department of Intensive Care Medicine Medical Faculty RWTH Aachen University Hospital RWTH Aachen Aachen Germany; 2 Department of Anaesthesiology St. Elisabeth Hospital Jülich Germany; 3 Department of Anaesthesiology Franziskushospital Aachen Germany; 4 Department of Medical Statistics Medical Faculty RWTH Aachen University Hospital RWTH Aachen Aachen Germany

**Keywords:** intensive care, outcome improvement, sepsis, sepsis bundle compliance, SSC, tele-ICU, telemedicine

## Abstract

**Background:**

Sepsis is a major health care problem with high morbidity and mortality rates and affects millions of patients. Telemedicine, defined as the exchange of medical information via electronic communication, improves the outcome of patients with sepsis and decreases the mortality rate and length of stay in the intensive care unit (ICU). Additional telemedicine rounds could be an effective component of performance-improvement programs for sepsis, especially in underserved rural areas and hospitals without ready access to critical care physicians.

**Objective:**

Our aim was to evaluate the impact of additional daily telemedicine rounds on adherence to sepsis bundles. We hypothesized that additional telemedicine support may increase adherence to sepsis guidelines and improve the detection rates of sepsis and septic shock.

**Methods:**

We conducted a retrospective, observational, multicenter study between January 2014 and July 2015 with one tele-ICU center and three ICUs in Germany. We implemented telemedicine as part of standard care and collected data continuously during the study. During the daily telemedicine rounds, routine screening for sepsis was conducted and adherence to the Surviving Sepsis Campaign’s 3-hour and 6-hour sepsis bundles were evaluated.

**Results:**

In total, 1168 patients were included in this study, of which 196 were positive for severe sepsis and septic shock. We found that additional telemedicine rounds improved adherence to the 3-hour (Quarter 1, 35% vs Quarter 6, 76.2%; *P*=.01) and 6-hour (Quarter 1, 50% vs Quarter 6, 95.2%; *P*=.001) sepsis bundles. In addition, we noted an increase in adherence to the item “Administration of fluids when hypotension” (Quarter 1, 80% vs Quarter 6, 100%; *P*=.049) of the 3-hour bundle and the item “Remeasurement of lactate” (Quarter 1, 65% vs Quarter 6, 100%, *P*=.003) of the 6-hour bundle. The ICU length of stay after diagnosis of severe sepsis and septic shock remained unchanged over the observation period. Due to a higher number of patients with sepsis in Quarter 5 (N=60) than in other quarters, we observed stronger effects of the additional rounds on mortality in this quarter (Quarter 1, 50% vs Quarter 5, 23.33%, *P*=.046).

**Conclusions:**

Additional telemedicine rounds are an effective component of and should be included in performance-improvement programs for sepsis management.

## Introduction

Sepsis is the most-frequent cause of morbidity and mortality in most intensive care units (ICUs) worldwide. The incidence of sepsis is increasing due to the high prevalence of severe comorbidities in the ageing population and the growing bacterial drug resistance [1]. In Germany, the number of cases of sepsis increased by 5.7% annually from 2007 to 2013. Although the mortality rate is constantly decreasing worldwide, it is still high (30%-50%) [2]. A recently published observational study by the SepNet Critical Care Trials Group showed that application of the new Sepsis-3 definition [3] led to higher rates of ICU and in-hospital mortality (>50%) in patients with severe sepsis or septic shock than in those diagnosed using the previous definition [4]. Early detection of sepsis followed by early initiation of adequate management can significantly improve the outcome in patients with sepsis [5].

The European Society of Intensive Care Medicine and the Society of Critical Care Medicine published the Surviving Sepsis Campaign (SSC) guidelines for the management of severe sepsis and septic shock, with an aim to reduce the mortality of sepsis by 25% in 5 years [5,6,7]. A large body of evidence indicates that adherence to clinical practice guidelines and compliance with sepsis bundles are associated with reduced ICU length of stay, low mortality rates, and improved patient outcome [8,9]. In addition, the SSC guidelines specifically emphasize the need for performance-improvement programs for sepsis [5] and recommend an interdisciplinary approach to sepsis management, protocol development, and implementation; evaluation of targeted metrics; continuous data collection; and continuous feedback to allow constant performance improvement. Although the details may vary among different improvement programs, the common goal is to improve compliance with sepsis bundles and clinical practice guidelines [5,6,7]. However, recent studies showed that compliance with sepsis bundles is still low [8,10,11].

Telemedicine, defined as the exchange of medical information via electronic communication, can be used to improve the availability and quality of medical care. In the intensive care setting, telemedicine may improve early detection and appropriate treatment of severe sepsis and septic shock [12]. Telemedicine facilitates direct interaction among intensive care providers as an around-the-clock service over long distances and physicians who care for critically ill patients (intensivist to physician). Telemedicine enables critical decision support by exchanging clinical data in real time [13]. Recently, a systematic review demonstrated that telemedicine can improve the outcome of critically ill patients and decrease ICU mortality and length of stay [14]. However, no study has thus far determined whether additional telemedicine rounds could be an effective component of performance-improvement programs for sepsis, especially in underserved rural areas and hospitals without ready access to critical care physicians. Our objective was to evaluate the impact of additional daily telemedicine rounds via an audio-video system on the adherence to 3-hour and 6-hour sepsis bundles in three ICUs in Germany. We hypothesized that additional telemedicine support will increase adherence to sepsis guidelines and improve the detection rates of severe sepsis and septic shock.

## Methods

### Study Design and Oversight

We performed an 18-month retrospective, observational, multicenter study in three ICUs within three hospitals in North Rhine Westphalia (Germany). The study started in January 2014 and ended in June 2015 (18 months). We included only adult, nonpregnant patients aged ≥18 years, with no advanced care directives that limited life-saving care in our analysis. The study was reviewed and approved by the local institutional ethics board of the University Hospital RWTH Aachen (262/13). We implemented telemedicine as part of standard of care; as the analysis was performed retrospectively, the ethics board waved the need for informed consent.

### Characteristics of Intensive Care Units

One ICU (A), focusing on neurosurgery and general surgery, was located at a University Hospital and staffed by an intensivist around the clock. Two interdisciplinary ICUs (B and C) were located in community hospitals. ICU B was staffed by a general anesthesiologist and an internal specialist on weekdays. After regular day shifts, on-call personnel or anesthesia house staff was responsible for the treatment of ICU patients during the night and on weekends. ICU C was staffed by physicians of the Department of Internal Medicine and by anesthesiologists during regular day shifts; during the night and on weekends, an anesthesiologist was on-call.

### Tele-ICU Center and Telemedicine Infrastructure

**Figure 1:**
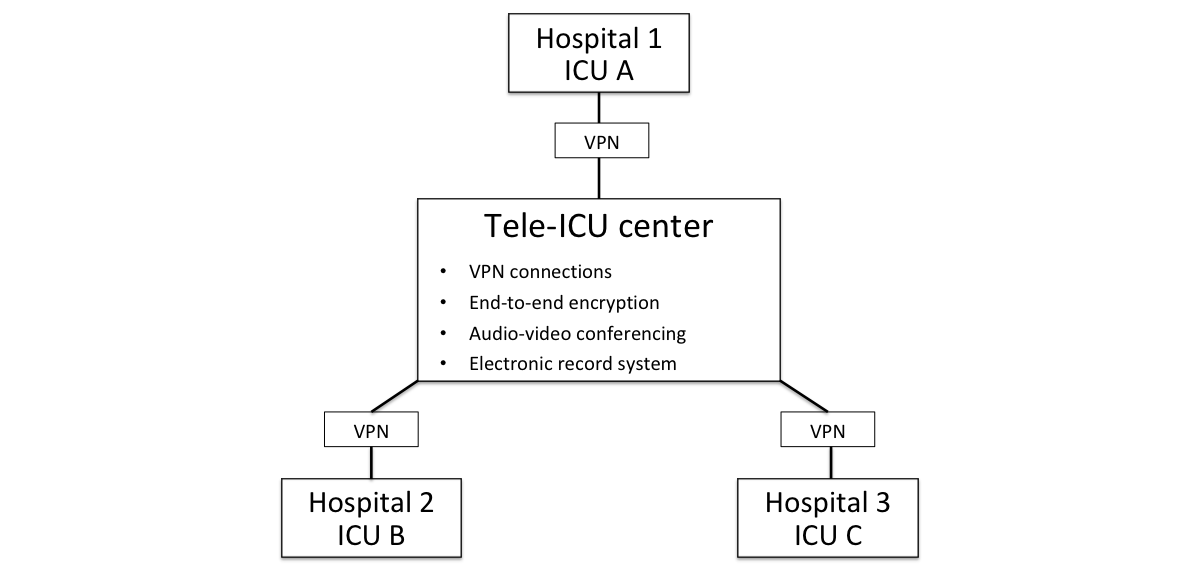
Outline of the telemedicine center and the participating ICUs. ICU: intensive care unit, VPN: virtual private network.

The Telemedicine Center at the University Hospital RWTH Aachen was the leading center for this study. As a preparatory measure, both the tele-ICU system and the electronic health record (*FallAkte*; Soarian Integrated Care, Siemens, Munich, Germany) were customized for use in the ICUs and the tele-ICU. All participating personnel received standardized information including the SSC guidelines and relevant literature [3,4,5]. Prior to the beginning of the study, a permanently installed tele-ICU system was implemented at the Telemedicine Center in the University Hospital RWTH Aachen and mobile tele-ICU systems were installed at the participating ICUs (Figure 1). A secure encrypted site-to-site virtual private network connection was established. The Telemedicine Center and participating ICUs were equipped with identical audiovisual transmission equipment (Cisco Systems, Inc, San José, CA). In addition, the Telemedicine Center received a workstation, multiple monitors, and a video system (Cisco Systems, Inc). The participating ICUs were equipped with a mobile audio-video system that could be taken to the patient’s room during rounds. Videoconferencing allowed two or more units to communicate simultaneously via a two-way high-resolution video and audio transmission. The video system included an option for closeup zoom that allowed detailed examination of both the patient and the bedside equipment. To enable exchange of medical data, the secure data-protection platform *FallAkte* was established. Accordingly, the system integrated a “store and forward” technique for structured data combined with real-time audio-visual telemedicine rounds, through which the vital signs were measured. After checking for missing data, all patient data were anonymized and exported to the University Hospital’s research database. Figure 1 outlines the structure of the Telemedicine Center and the participating ICUs.

The Telemedicine Center was staffed with an intensive care physician from 7:30 AM to 4 PM on weekdays and from 9 AM to 5:30 PM on weekends. Telemedicine rounds were performed on a daily basis. If required, additional rounds were offered after core hours and at night by the intensive care consultant on duty, ensuring accessibility of telemedical consultations around the clock.

Medical documentation in two ICUs was entirely paper based before the study started. To allow standardized documentation, project-specific templates were designed (Adobe Life Cycle, San José, CA) and filled in by physicians in the Telemedicine Center and ICUs during daily rounds. The templates covered basic demographic data of patients and summarized diagnoses, performed procedures, and therapies. Furthermore, the severity of disease and functional limitations of the patient were assessed by the Simplified Acute Physiology Score II (SAPS II) and Sepsis-related Organ Failure Assessment (SOFA) scales [13]. A systematic checklist for daily infection assessment was used. During rounds, both case presentation and assessment were performed by the local physician and the intensivist in the tele-ICU; the diagnostic or therapeutic interventions were discussed, and direct feedback regarding sepsis management was provided. Typically, conscious patients or their relatives were present during the telemedicine rounds.

All telemedicine rounds were observed and documented by a research assistant. The following items were documented: recommendations for diagnosis and therapy of sepsis, adjustments of sepsis management, details regarding antibiotic therapy (continued, evaluated, changed, or terminated), scoring of SAPS II and SOFA, duration of ICU length of stay (LOS), and duration of ICU LOS after sepsis diagnosis.

### Definitions

The applied definitions of sepsis, severe sepsis, and septic shock are outlined in Multimedia Appendix 1 [7,15].

### Evaluation of Adherence to 3-Hour and 6-Hour Sepsis Bundles in Patients With Sepsis

We continuously extracted data during the study and follow-up to evaluate whether sepsis management fulfilled the requirements of the 3-hour and 6-hour sepsis bundles. Time “0” was defined as the time when the attending physician diagnosed sepsis. Items of the 3-hour and 6-hour bundles for patients with severe sepsis and septic shock were adapted from the SSC standard sepsis resuscitation guidelines. The 3-hour bundle includes the following recommendations: measurement of lactate levels, blood cultures obtained prior to administration of antibiotics, administration of broad spectrum antibiotics, and administration of 20 mL/kg crystalloid fluid for hypertension or lactate levels ≥4 mmol/L. The 6-hour bundle consisted of the following core items: application of vasopressors for persistent hypotension (mean arterial pressure <65 mmHg) despite initial fluid resuscitation, assessment of central venous pressure and central venous oxygen saturation if hypotension persisted despite initial fluid administration or when initial lactate levels were ≥4 mmol/L, and remeasurement of lactate levels at initial elevation. The second bundle was also applied to patients with persistent hypotension or high lactate levels within the 6-hour period.

For evaluation of adherence to the 3-hour and 6-hour sepsis bundles, we assigned a “yes” rating to patients with sepsis if all core items of the respective bundle were executed within 3-hours or 6-hours after time “0”; otherwise, a “no” rating was assigned.

### Statistical Analysis

Categorical data are presented as frequency and percentage. Frequencies of categorical data were compared between groups by the Fisher exact test. Continuous variables are expressed as mean values (SD). Differences in continuous data between groups were analyzed by the *t* test, assuming unequal variances. Statistical tests were two-tailed, and values of *P*<.05 were considered significant. SAS software, version 9.4 (SAS Institute Inc, Cary, NC), and GraphPad Prism software, version 6.0 (GraphPad Software, La Jolla, CA), were used for statistical analyses.

## Results

The study included 1168 patients who received 4569 telemedicine rounds in addition to their daily rounds at the local ICUs. Physicians at the 3 ICUs performed 4373 infections and sepsis screenings. The telemedicine rounds were performed routinely in the morning when it was most convenient for the staff of the participating ICU. There were rarely emergency visits outside the core visit times. Emergency telemedicine visits were infrequent, especially for patients with acute respiratory distress syndrome, craniocerebral trauma, or septic shock, and the involved staff scheduled these events, if needed. During the study, the average visiting time was 4.67 (SD 2.55) minutes. The duration of the telemedicine rounds was comparable among patients during the course of the study (range of mean: 4.18-4.63) with similar variation (range of SD: 2.27-2.82).

Overall, we observed a decrease in mortality from 50% in Quarter 1 (January 1, 2014 to March 31, 2014) to 33.33% in Q6 (April 1, 2015 to June 30, 2015; *P*=.35) in patients with severe sepsis and septic shock and a comparable degree of severity among patients, as per the SAPS II and SOFA score (Tables 1 and 2). Due to a higher number of patients with sepsis in Quarter 5 (N=60) than in other quarters, we observed stronger and statistically significant effects of the additional rounds on mortality in this quarter (Quarter 1, 50% vs Quarter 5, 23.33%, *P*=.046). ICU LOS after diagnosis of severe sepsis and septic shock remained unchanged over the observation period. We presented the number of scorings, number of therapeutic recommendations made, and the overall number of sepsis detections per quarter in Table 1. The mean age of the included patients was 64.91 (SD 17.09) years, and 584 of 1168 patients (50%) were male. In total, 196 patients showed positive results for severe sepsis (N=95) or septic shock (N=101) during the study period and were included in our analysis. Table 2 summarizes the patient characteristics and provides details on ICU LOS and ICU LOS after diagnosis of sepsis for Quarters 1, 5, and 6.

**Figure 2:**
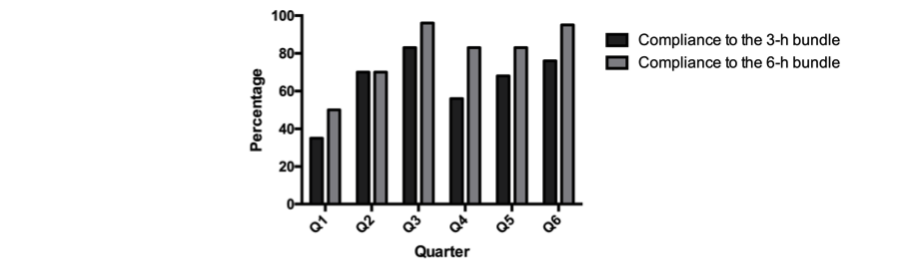
Impact of additional telemedicine rounds on adherence to sepsis bundles.

**Figure 3:**
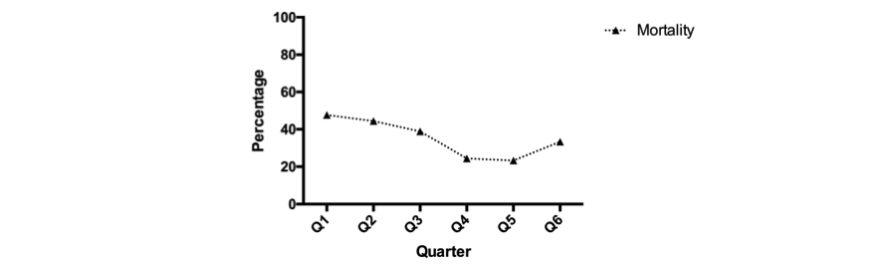
Impact of additional telemedicine rounds on mortality.

Our primary objective was to evaluate the impact of additional daily telemedicine rounds on adherence to the sepsis bundles in order to determine whether additional telemedicine rounds are an effective performance-improvement strategy for sepsis management. We found that additional telemedicine rounds had a statistically significant effect on adherence to the 3-hour (*P*=.01) and the 6-hour (*P*=.001) sepsis bundles. In addition, we observed an increase in adherence to the item “Administration of fluids when hypotension” (*P*=.049) with the 3-hour bundle and to the item “Remeasurement of lactate” (*P*=.003) with the 6-hour bundle. All results of the impact of telemedicine rounds on adherence to sepsis bundles are presented in Table 3 and Figure 2. Figure 3 illustrates the change in mortality among patients diagnosed with sepsis.

**Table 1:** Study characteristics.

Study characteristics	Quarter 1, n (%)	Quarter 2, n (%)	Quarter 3, n (%)	Quarter 4, n (%)	Quarter 5, n (%)	Quarter 6, n (%)	Total (N)
Telemedicine rounds	541 (11.84)	596 (13.04)	953 (20.86)	812 (17.77)	1074 (23.50)	593 (12.98)	4569
Infection and sepsis screenings	424 (9.70)	591 (13.51)	990 (22.64)	775 (17.72)	1030 (23.55)	563 (12.87)	4373
Scorings (SAPS^a^ II and SOFA^b^)	541 (11.84)	596 (13.04)	953 (20.86)	812 (17.77)	1074 (23.51)	593 (12.98)	4569
Diagnostic recommendations	90 (12.21)	77 (10.45)	162 (21.98)	108 (14.65)	203 (27.54)	97 (13.16)	737
Therapeutic recommendations	111 (9.28)	82 (6.85)	285 (23.82)	202 (16.89)	363 (30.35)	153 (12.79)	1196
Total 3-h and 6-h bundles	20 (10.20)	18 (9.18)	36 (18.37)	41 (20.92)	60 (30.61)	21 (10.71)	196
Severe sepsis or septic shock detections	20 (10.20)	18 (9.18)	36 (18.37)	41 (20.92)	60 (30.61)	21 (10.71)	196
Mortality	10/20 (50)	8/18 (44.44)	14/36 (38.88)	10/41 (24.39)	14/60 (23.33)	7/21 (33.33)	63

^a^SAPS: Simplified Acute Physiology Score.

^b^SOFA: Sepsis-related Organ Failure Assessment.

**Table 2:** Characteristics of patients with severe sepsis and septic shock.

Patient characteristics	Initiation of telemedicine rounds, Quarter 1 (n=20)	After implementation in Quarter 5 (n=60)	After implementation in Quarter 6 (n=21)	*P* value	
				Comparison between Q1 and Q5	Comparison between Q1 and Q6
Patients with severe sepsis, n (%)	10 (50.0)	34 (56.7)^a^	6 (28.6)^b^	.62	.21
Patients with septic shock, n (%)	10 (50.0)	26 (43.3)^a^	15 (71.4)^b^	.62	.21
Mortality, n (%)	10 (50.0)	14 (23.3)^a^	7 (33.3)^b^	*.* 046	.35
LOS^c^ ICU^d^ (days), mean (SD)	18.2 (21.6)	15.65 (15.5)^a^	19.48 (21.4)^b^	.63	.85
LOS ICU after diagnosis of sepsis (days), mean (SD)	15.65 (21.1)	13.22 (13.9)^a^	16.76 (20.7)^b^	.64	.87
SAPS^e^ II, mean (SD)	44.35 (12.1)	44.16 (16.4)^a^	45.76 (14.4)^b^	.96	.74
SOFA^f^, mean (SD)	7.7 (3.1)	7.18 (3.9)^a^	7.52 (3.6)^b^	.55	.86

^a^Comparison between Q1 and Q5.

^b^Comparison between Q1 and Q6.

^c^LOS: length of stay.

^d^ICU: intensive care unit.

^e^SAPS: Simplified Acute Physiology Score.

^f^SOFA: Sepsis-related Organ Failure Assessment.

**Table 3:** Impact of telemedicine rounds on adherence to sepsis bundles.

Parameters		Quarter 1^a^ (N=20), n (%)		Quarter 6^b^ (N=21), n (%)		*P* value	
Compliance to the 3-h bundle		7 (35.0)		16 (76.2)		.01	
Compliance to the 6-h bundle		10 (50.0)		20 (95.2)		.001	
**Components or target values of the 3-h bundle**							
	Serum lactate measurement		20 (100.0)		21 (100.0)		>.99
	Blood cultures before antibiotics		11 (55.0)		16 (76.2)		.20
	Administration of antibiotics within the first 3 h		19 (95.0)		21 (100.0)		.49
	Administration of fluids during hypotension		16 (80.0)		21 (100.0)		.049
	Administration of vasopressors when indicated		18 (90.0)		20 (95.2)		.61
	CVP^c^ >8 mmHg		16 (80.0)		19 (90.5)		.34
	ScvO_2_^d^ >70%		5 (25.0)		9 (42.9)		.33
**Components or target values of the 6-h bundle**							
	Administration of vasopressors when indicated		18 (90.0)		20 (95.2)		.61
	Assessment of CVP when indicated		16 (80.0)		19 (90.5)		.41
	Assessment of ScvO_2_ when indicated		4 (20.0)		9 (42.9)		.18
	Remeasurement of lactate		13 (65.0)		21 (100.0)		.003

^a^Initiation of telemedicine rounds.

^b^After implementation of additional rounds.

^c^CVP: central venous pressure.

^d^ScvO_2_: central venous oxygen saturation.

## Discussion

To the best of our knowledge, this is the first study to evaluate whether additional telemedicine rounds should be included in performance-improvement strategies for sepsis management, especially in underserved rural areas and hospitals without ready 24-hour access to critical care physicians. Of the 1168 patients included in this study, 196 were positive for severe sepsis and septic shock. The proportion of patients with severe sepsis and septic shock was in the expected range and in line with the findings of recent epidemiologic surveys [16,17,18]. We found that additional telemedicine rounds had a significant effect on the adherence to the 3-hour and the 6-hour sepsis bundles and improved adherence to current clinical practice guidelines and patient care in sepsis management. In addition, we observed an increase in adherence to the item “Administration of fluids when hypotension” of the 3-hour bundle and the item “Re-measurement of lactate” in the 6-hour bundle. Moreover, we observed a decrease in mortality among patients with severe sepsis and septic shock and a comparable degree of severity among all patients, as per the SAPS II and SOFA scores. The ICU LOS after diagnosis of severe sepsis and septic shock remained unchanged over the observation period. Due to a higher number of patients with sepsis in Quarter 5 (N=60) than in other quarters, we observed stronger effects of the additional rounds on mortality in this quarter. Our findings are consistent with those of other studies or health care approaches investigating sepsis management in the intensive care medical setting [19,20,21].

Additional telemedicine rounds with standardized daily sepsis screening significantly improved guideline adherence. Near real-time feedback in an intensivist-driven tele-ICU system is an effective performance-improvement strategy for rapid implementation of evidence-based practice to achieve improved quality of care. In Germany, the foundation of telemedicine was laid when the first guideline on telemedicine in intensive care was published by the Association of the Scientific Medical Societies (Arbeitsgemeinschaft der Wissenschaftlichen Medizinischen Fachgesellschaften) and the German Society of Anaesthesiology and Intensive Care Medicine (Deutsche Gesellschaft für Anästhesiologie und Intensivmedizin) [22]. Previous studies showed that performance-improvement programs are associated with increased adherence to resuscitation and management of sepsis bundles, with reduced mortality in patients with sepsis, severe sepsis, or septic shock [23]. As a continuous effort, all involved stakeholders should aim to implement telemedicine nationwide as a part of the daily routine standard of care. Constant measurement of the influence of telemedicine on quality indicators for intensive care is important, as demonstrated in this study.

The implementation of telemedicine is important in light of recent findings of Faine and colleagues [24], who showed that interhospital transfers delay appropriate treatment for patients with severe sepsis and septic shock. Telemedicine can improve on-site quality of care by shared expertise, as it is a viable alternative to urgent patient transfers to university centers. As recently demonstrated by Pannu et al [25], telemedicine comanagement slightly increased patient transfers to high-level centers. However, our findings are consistent with those reported by two systematic reviews and meta-analyses and multiple study reports, which revealed that telemedicine is associated with low ICU mortality [26,27,28,29] and low ICU LOS [26,29,30]. Furthermore, Lilly et al emphasized that telemedicine has the potential to improve adherence to ICU best practices, reduce response times to alarms, and encourage the use of performance data in the ICU [27].

Owing to its explorative nature, this study had a few limitations that should be considered when interpreting our results. First, we only included a small number of participating hospitals, resulting in a small numbers of patients. However, these are preliminary results and provide crucial data for subsequent large-scale trials. Second, the level of acceptance, an important factor for the success for telemedicine, especially in the ICU, was not measured systematically in this study. Therefore, we could not estimate the possible influence of telemedicine as a cofounding factor. However, personal communication during the telemedicine rounds revealed positive feedback and a high level of acceptance by the participating physicians, nurses, patients, and their relatives. Our experience is in line with the findings of other telemedicine studies [30,31] that reported high levels of staff acceptance of telemedicine and tele-ICU coverage. Similar to other medical fields [32], the overall attitude toward telemedicine and eHealth in the intensive care setting was consistently positive and in favor of the technology. This aspect should be assessed as a possible confounding factor in rigorously planned, methodological, high-quality studies in the future. Third, the geographical and time-specific design may limit the extrapolation of our results to other medical centers and patients. We believe that an advanced technical setup will further improve the acceptance of telemedicine by, for example, reducing the workload of documentation. Automatic data capture by export of the international Health Level 7 standard into an active tele-ICU system with automatic calculation of disease severity or sepsis alerts may improve care beyond the results of this study. Fourth, it is important to carefully consider the generalizability of data obtained from a retrospective study such as this study. However, we continuously documented sepsis onset and sepsis bundle compliance throughout our study. This approach reduced difficulties associated with the retrospective identification of the time of sepsis onset as “time zero” for the evaluation of sepsis bundle compliance. This approach might limit the explanatory power of effect size and causality of the tele-ICU concept, but offers a relevant benefit of positive outcomes for patients enrolled in such an implementation study. The last limitation was with regard to some methodological characteristics of our study design. Our retrospective data analysis was based on the evaluation of six consecutive quarters after implementing additional telemedicine rounds as a part of standard care for ICU patients. Patients were included continuously over the course of the six quarters and the number of rounds and total number of infection and sepsis screenings were recorded per quarter. However, in one ICU, some patients received additional rounds in two subsequent quarters. As such, we could not present some data items specific to one quarter, which limited our ability to perform a pre-post comparison or patient-level logistic regression analysis including adjustment for patient demographics, comorbidities, or sepsis severity. However, we believe that our findings, which showed a significant advance in sepsis management over the course of six quarters with telemedicine support, supports the implementation of additional telemedicine rounds as a successful performance-improvement strategy for sepsis management.

## Appendix

Multimedia Appendix 1 Definitions of sepsis, severe sepsis or septic shock.

## Abbreviations

CVP: central venous pressure
ICU: intensive care unit
LOS: length of stay
ScvO_2_: central venous oxygen saturation
SOFA: Sepsis-related Organ Failure Assessment
SAPS: Simplified Acute Physiology Score
SSC: Surviving Sepsis Campaign
