# Supplementary material for: Additional Telemedicine Rounds as a Successful Performance-Improvement Strategy for Sepsis Management: Observational Multicenter Study
Source: J Med Internet Res. 2019 Jan 15;21(1):e11161. doi: 10.2196/11161 (PMC6350091; doi:10.2196/11161)
Supplement: Multimedia Appendix 1 [file jmir_v21i1e11161_app1.pdf]

Multimedia Appendix 1 – Definitions of sepsis, severe sepsis or septic shock [7,15]

**Sepsis**	Presence (probable or documented) of infection with systemic manifestation:<br>**General variables:**<br>Fever >38.3°C, hypothermia < 36°C, heart rate > 90 bpm or more than two SD above the normal value for age, tachypnea, altered mental status, significant oedema or positive fluid balance >20 ml/kg over 24 hours, hyperglycaemia with a plasma glucose > 140 mg/dl or 7.7mmol/L in the absence of diabetes mellitus<br>**Inflammatory variables:**<br>Leukocytosis (WBC count > 12,000 μl^{-1}), leukopenia (WBC count < 4,000 μl^{-1}), normal WBC with greater than 10% immature forms, plasma C-reactive protein more than two SD above the normal value, plasma procalcitonin more than two SD above the normal value<br>**Hemodynamic variables:**<br>Arterial hypotension (SBP <90 mmHg, mean BP <65 mmHg, or reduction in SBP >40 mmHg from baseline) persisting for at least 1 hour, despite adequate fluid resuscitation<br>**Organ dysfunction variables:**<br>Arterial hypoxemia (PaO_2/FiO_2 <300), acute oliguria (urine output <0.5 ml kg^{-1}/h^{-1} for at least 2 h) creatinine increase ≥0.5 mg dL^{-1}, coagulation abnormalities (INR >1.5 or activated partial thromboplastin time >60 s), ileus (absent bowel sounds), thrombocytopenia (platelet count <100,000/μL), hyperbilirubinemia (plasma total bilirubin >4 mg dL^{-1} or 70 mmol L^{-1})<br>**Tissue perfusion variables:**<br>Hyperlactatemia (>3 mmol/L^{-1}), decreased capillary refill or mottling
**Severe sepsis**	Sepsis plus sepsis-induced organ dysfunction or tissue hypoperfusion:<br>Altered mental state<br>Arterial hypoxemia (PaO_2/FiO_2 < 300)<br>Acute kidney injury (urine output < 0.5 ml/kg/h for at least 2h or creatinine increase > 0.5 mg/dL)<br>Thrombocytopenia (platelet count < 100.000/µl)<br>Coagulopathy (INR > 1.5 or aPTT > 60 s)<br>Hyperbilirubinemia (total plasma bilirubin > 2 mg/dl)<br>Hyperlactatemia (> 1 mmol/L)<br>Sepsis-induced hypotension<br>Decreased capillary refill or mottling
**Septic shock**	Sepsis-induced hypotension (SBP < 90 mmHg or MAP <

	70 mmHg or SBP decrease > 40 mmHg) persisting despite adequate fluid resuscitation

Abbreviations C: Celsius; bpm: beats per minute; SD: standard deviation; ml: milliliter; kg: kilogram; mg: milligram; dl: deciliter; mmol: millimol; L: liter; WBC: white blood cell; µl: microliter; SBP: systolic blood pressure; mmHg: millimeter of mercury; MAP: mean arterial pressure; aPTT: activated partial thromboplastin time; SBP: systemic blood pressure; MAP: mean arterial pressure; PaO_2/FiO_2: ratio of arterial oxygen partial pressure to fractional inspired oxygen; INR: international normalized ratio, INR: international normalized ratio.
